# Bi-directional plasticity: Rotifer prey adjust spine length to different predator regimes

**DOI:** 10.1038/s41598-017-08772-7

**Published:** 2017-08-31

**Authors:** Huan Zhang, Johan Hollander, Lars-Anders Hansson

**Affiliations:** Department of Biology, Aquatic Ecology, Lund University, Lund, Sweden

## Abstract

Numerous prey organisms, including many rotifers, exhibit inducible defensive plasticity, such as spines, in response to predators. Here, we test the hypothesis that prey modify their defence response to different predator sizes with a bi-directional adjustment in spine length. First, we show experimentally, that large-sized predators induce a reduction in prey spine length. Second, we conducted a complementary field monitoring study showing that the spine length of the prey rotifer *Keratella cochlearis* changed in opposite directions, in response to the shift in dominance between small-sized and large-sized predators. Third, in order to test the generality of our novel findings, we conducted a meta-analysis covering a wide array of rotifer prey taxa, strengthening the conclusions from our experimental and field studies. Hence, by combining evidence from experiments and studies in the field with a meta-analysis, we, for the first time, demonstrate that rotifer prey distinguish between predators and adjust their protective spine length accordingly, i.e. rapidly adjust spine length to escape either below or above the dominant predator’s gape size window. In a broader perspective, our conclusions advance our knowledge on observed spatial and temporal variations in protective morphologies among prey organisms.

## Introduction

Organisms in the wild simultaneously experience and handle a wide array of threats that may fluctuate in intensity both spatially and temporally. Accordingly, many prey organisms are able to show plasticity in behavior and morphology^1–3^, or life-history traits, such as age at maturity and clutch size^4^. Most studies on predator threats focus on how a prey responds to one predator, although different predators dominate during different times of the year, i.e. prey have to adapt to different, and sometimes multiple predator regimes both spatially and temporally^5, 6^. Predators are, on the other hand, constrained by their prey-size choices, for example by gape-size limitations and it has been repeatedly demonstrated that, in order to reduce the predation rate, prey respond by growing larger than the gape size limit of the predators^7, 8^. However, it may also be adaptive for a prey to escape from predation through avoiding the lower range of a predator’s gape size, although this has rarely been demonstrated^9^. Hence, different predator taxa may select for different slopes and end points of the prey’s reaction norm, suggesting that the adaptive value of becoming larger or smaller differs spatially and temporally with the gape-size optimum of the dominant predator.

In most freshwater ecosystems on Earth, a common and often dominant group of organisms are rotifers which range in length from 0.05 mm to over 2 mm^10^, although most of them are around 0.05–0.5 mm long. Many rotifer taxa are vulnerable prey and possess inducible defensive morphological characters, such as spines, in response to kairomones from predators^10–14^. Most studies are, however, performed on relatively small predators, where it may be adaptive for the prey rotifers to develop longer spines in order to increase handling time. However, in the wild prey are exposed to predators with different gape-size optima and having long spines may not be adaptive if the predator is very large. Instead, the prey should then be expected to reduce their spine length in order to escape below the gape-size optimum of the predator. Hence, prey rotifers have to cope with predators ranging in size from approximately two-times larger, such as *Asplanchna* spp., to more than 100 times their own size, e.g. fish larvae^15^. Although growing longer spines may be adaptive towards small predators, they may instead be maladaptive when large predators dominate. This suggests that although it may be adaptive to develop longer spines when small predators dominate^10^, reduced spine length should allow escaping from the optimal gape-size of larger predators. A few previous studies have actually noted a reduced spine length in prey rotifers when exposed to large invertebrate predators, such as the ostracod *Cypris pubera* and the notonectid insect *Buenoa fuscipennis* ^16, 17^. Therefore we aim to test the hypothesis that prey defence response is predator size-specific with bi-directional responses in spine length depending on whether the prey escapes above or below the gape-size limits of the predator. We tested this hypothesis experimentally, in a field monitoring study as well as in a meta-analysis. Based on a synthesis of these three approaches we demonstrate, for the first time, that the induction of protective spines in a common invertebrate prey is bi-directional and driven by the dominant predator.

## Material and Methods

### Experiment

That rotifers, such as *Keratella cochlearis*, develop longer spines in the presence of small predators has been shown repeatedly^10^. Therefore, the aim of our experiment was to assess the effects of kairomones on spine length, from relatively large predators, along a body size gradient, including the cyclopoid copepod *Cyclops* sp. (mean length 3.8 mm), the insect larva, *Chaoborus flavicans* (mean length 10 mm), and small fish *Paracheirodon innesi* (mean length 23 mm). *C. flavicans* and *Cyclops* sp. were collected in a small fish-free pond in Lund, southern Sweden. Together with the small fish *P. innesi*, they constitute a size gradient of predators but are all more than 10 times larger than the prey (see Supporting information Table S1 for more information about predator size). Lake water and *K*. *cochlearis* were collected from Lake Krankesjön (N55°42′27″, E13°27′58″) on 15 March 2016. The culture medium (hereafter, called basic medium) comprised lake water filtered through a 20-μm mesh and then incubated for two days at 15 °C. Predator-conditioned medium was prepared by culturing three small fish (*P*. *innesi*) in one litre basic medium, 40 individuals of copepods (*Cyclops* sp.) in 100 ml basic medium and 30 individuals of *Chaoborus* (*C*. *flavicans)* in 300 ml basic medium, respectively, for at least 20 hours. Each predator group was fed with *K*. *cochlearis* and *Polyarthra* sp. and each predator-conditioned medium was then filtered through a Whatman GF/C (1.2 μm) glass microfiber filter before use in the experiment. Concentrated rotifer populations were filtered by using 200-μm mesh to eliminate potential predators (e.g. Copepods and *Asplanchna*) and mixed thoroughly with basic medium. The mixed *K*. *cochlearis* and basic medium (150 ml each) was equally filled into thirty-two (8 replicates × 4 treatments) 250-ml glass bottles as starting cultures. The effects of predator-conditioned medium of these three predators on the spine length of *K*. *cochlearis* were tested by the daily addition of 5 ml of predator-conditioned medium to each predator treatment, and the same amount of basic medium was added to the control treatment. The cultures were maintained at 15 °C at a 14:10 h, light: dark cycle. 5 ml of an algal slurry (90% of *Scenedesmus* sp.) at a concentration of 1 mg L^−1^ chlorophyll-*a* were added to each replicate every fifth day as food for rotifers. At the start of the experiment and after 12 days, 25 ml samples were taken from each replicate for the analysis of *K*. *cochlearis* posterior spine length and total length. Samples were preserved with Lugol’s acid solution for later measurements according to the same method as used for the monitoring experiment described below. Posterior spine length of *K*. *cochlearis* from both the start and the end sampling were compared among treatments by using one-way ANOVAs, and Tukey’s HSD tests were used for multiple comparisons.

### Field study

The aim of the field monitoring study was to test whether there was a change in spine length in *K. cochlearis* during a period when there was a dominance shift between differently sized predators. In many temperate lakes, *K. cochlearis* is one of the most dominant rotifer species during spring and summer and is present throughout almost the whole year in Lake Krankesjön (Hansson *et al*., unpublished data), southern Sweden (N55°42′27″, E13°27′58″, see Hansson *et al*. 2007 for more information about the lake). *Asplanchna* (mean length 0.8 mm, considered to be a small-sized predator of *K*. *cochlearis*) is one of the most important predators, but in late May to July newly hatched fish (considered to be a large-sized predator on *K*. *cochlearis*) become the dominant predators of rotifers^15^. Since we were interested in the variation in size of the posterior spine of *K*. *cochlearis* during a period when there is a shift in the dominance of different sized predators, zooplankton samples were taken weekly from the beginning of May to late July 2013 in Lake Krankesjön. Ten litres of water were taken with a Plexiglas tube (length = 1.2 m, diameter = 70 mm) from the upper water column and were pooled in a bucket. 100 ml subsamples of the lake water were taken and preserved with Lugol’s solution for later measurement of *K*. *cochlearis* spine length and total length. Zooplankton were sampled by filtering ten litres of water through a 45-μm plankton mesh and preserved with Lugol´s solution for later *Asplanchna* abundance analysis at 100X magnification (Olympus CK40). *K*. *cochlearis* were measured in 25-ml Ütermöhl chambers. The posterior spine length and total length of 20 individuals of *K*. *cochlearis* were measured using an Olympus CK40 microscope at 400X magnification.

### Data collection and database for meta-analysis

We searched the biological literature for studies reporting on predator induced morphological defence in rotifers. The literature search was conducted by using ISI Web of Science and Scopus with the relevant keywords: rotife* AND defen*, rotife* AND morpholog* AND predat*, and by searching the cited literature in the obtained papers, as well in recent reviews (e.g. Gilbert, 2013, Lass and Spaak 2003^12, 18^). Studies were collected for analysis until 1 January 2015. The following criteria had to be met in order for a publication to be included in the analysis: (1) the defence-inducing treatment included an appropriate control which had not been exposed to either predators or kairomone from predators, (2) the paper included a report of the means, some measure of the variance and the sample sizes for the control and the treated experimental replicates, (3) quantitative measures of the effect of induced responses on morphology changes of the prey were included, and (4) the publication was written in English. Studies that did not meet our criteria were omitted from the data set.

The final database included 21 studies published between 1967 and 2014 (Supporting information Table S2) comprising 38 experimental results and 3 unpublished experimental results from our own experiment described above (Supporting information Table S2). The final database included 13 species of rotifer prey from 4 genera (Supporting information Table S2). When several measurements were made on the same individual (e.g. lorica length, lorica width or in different spines), only the result showing the largest difference between the control and the treated rotifer was used. When raw data were not available, data were extracted from figures using the software GetData Graph Digtizer version 2.26.0.20.

### Predator group

The final database included 17 species of predators (Supporting information Tables S1 and S2). We categorised the species into two size-based groups (“small-sized predator” and “large-sized predator”). When the size of the predator was not reported, we used taxonomic literature to obtain the body size (Supporting information Table S1). Since the size of predator is a continuous variable, there is no definitive size boundary between our large and small sized predators. However, in order to compare the effects from large and small predators, they were assigned into the two groups with a cut-off at 2.0 mm, which is about 10 times the size of most of the rotifer prey. Although we are aware of that this cut-off is arbitrary, we argue that the handling time for a predator 10 times larger than its prey may only be marginally affected by prey spines. The small-sized predator group consisted of six species from the genus *Asplanchna* and four copepod species, which are all smaller than 2 mm (Supporting information Table S1). The large-sized predator group consisted of seven species (three insects, one copepod, one flatworm, one ostracod and one fish species) with a size range from 2.2 mm to 23 mm (Supporting information Table S1).

### Meta-analysis

Meta-analysis is a powerful statistical tool for systematic, quantitative analysis of results from different independent studies but requires a consistent measure of effect size for testing a general hypothesis. The quantitative measure of induced morphological defences in rotifers for each experiment was expressed as a common unit, the effect size. We used the standardised difference between the means of the experimental group and control group, and calculated Hedges’*d* to quantify the weighted effect size of morphological plasticity:

$$d=\frac{({\bar{X}}_{E}-{\bar{X}}_{C})}{s}J$$

where ${\bar{X}}_{C}$ is the mean of the control group, ${\bar{X}}_{E}$ is mean of the experimental group, *s* is the pooled standard deviation of the control and experimental groups, and *J* is a correction term that removes small-sample-size bias^19, 20^. We used a random effect model, which means that we assume a random component of variation among effect sizes and bias-corrected 95% bootstrap confidence intervals^21^. The effect sizes were then calculated to assess the magnitude of the morphological defence effects^20^.

Subgrouping analyses by predator size groups were conducted to determine whether there was a significant effect in the direction and magnitude of the morphological defences in prey to different predator size groups. Consequently, a negative effect size indicates an induced reduction in spine length compared to the control, and a positive effect size indicates that longer spines were induced. Additionally, we calculated Rosenthal’s fail-safe number to test for publication bias^22^ for different size groups. Rosenthal’s fail-safe number gives the number of studies with zero effect that would be required to reject our stated hypotheses. If the number is high enough (>5 N + 10, where N is the number of experiments), the analysis can be considered to be robust with respect to publication bias. All analyses were conducted using the software OpenMEE Windows 8^23^.

## Results

### Experiment

**Figure 1:**
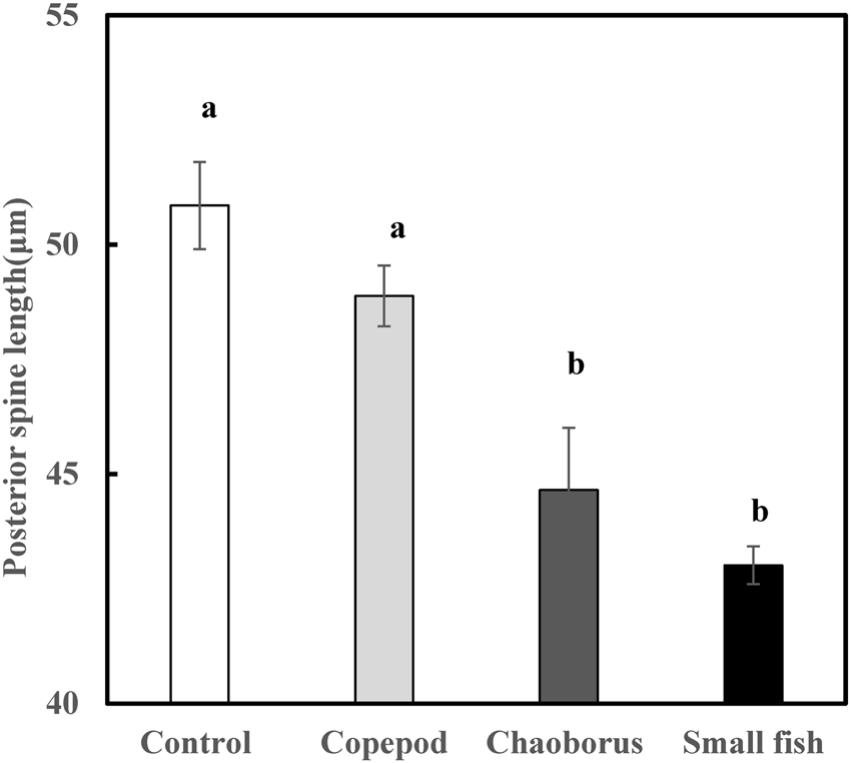
Posterior spine length of *Keratella cochlearis* after 12 days of exposure to kairomone from predator-free control aquaria and different predators, including the copepod *Cyclops* sp., the insect larvae *Chaoborus flavicans*, and small fish (*Paracheirodon innesi*). Values are means ± 1SE. Bars with different letters indicate that treatments are significantly different.

At the start of the experiment, the posterior spines of *K*. *cochlearis* were 58 ± 1.76 μm and there were no significant differences among the treatments (one-way ANOVA, *F*_3, 28_ = 1.15, p = 0.346). However, after exposure to the predator-conditioned medium for 12 days, the posterior spine length of *K*. *cochlearis* was significantly affected by kairomones from these predators (one-way ANOVA, *F*_3, 28_ = 11.81, p < 0.001, Fig. 1). The responses in the total length of *K*. *cochlearis* show similar patterns as the posterior spine length, to exposure to the predator-conditioned medium (Supporting information Fig. S1). Therefore, we focus on the responses of the posterior spine length. Separating the predator taxa showed that the posterior spines of *K*. *cochlearis* were largest in the control treatment, without any predator kairomones and showed no significant changes in response to kairomones from copepods, which was the smallest of the predators in the study (Tukey’s HSD, p = 0.748). However, kairomones from both *C*. *flavicans* and fish (*P*. *innesi*) significantly reduced the spine length of *K*. *cochlearis* by more than 15%, i.e. with more than 1% loss per day during the 12 days of the experiment (Tukey’s HSD, p < 0.001, Fig. 1). As only a few experimental studies exist on how large predators affect spine length in rotifers, these novel results were included in the meta-analysis (see Supporting information Table S2).

### Field study

**Figure 2:**
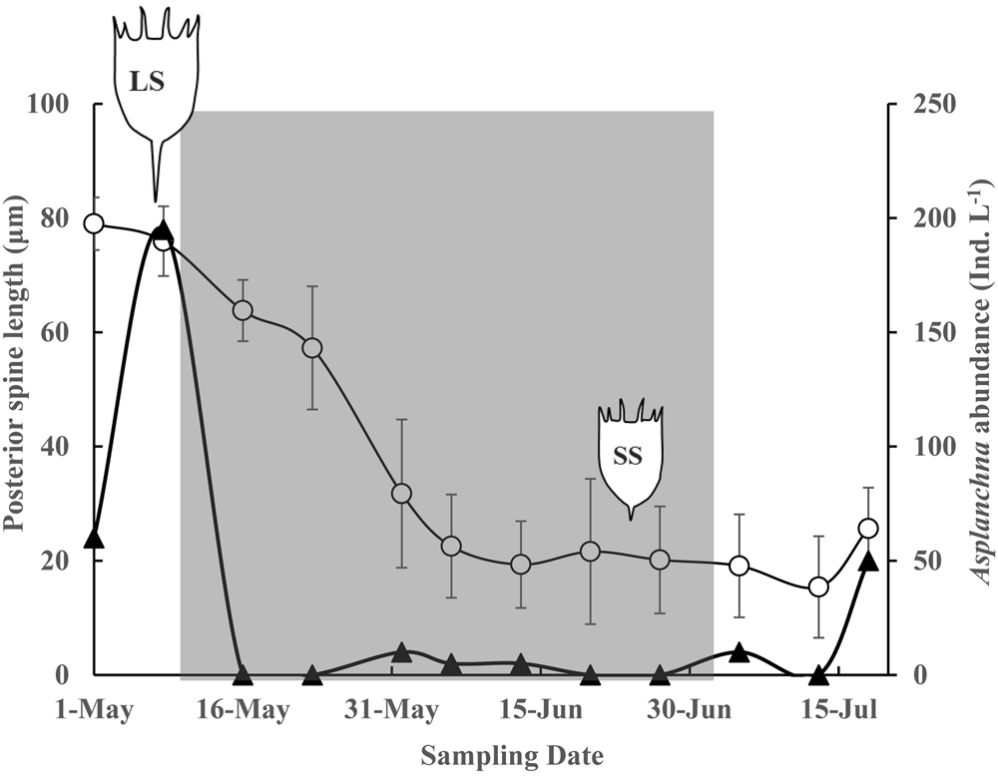
Posterior spine length variations in *Keratella cochlearis* (mean ± 1 SD; n = 20 individuals) and abundances of the small-sized predator *Asplanchna* from May to July 2013. The grey area indicates the period when newly hatched young-of-the-year fish feed on rotifers in Lake Krankesjön. Open circles represent posterior spine length of *K. cochlearis* (means ± 1 SD) and triangles represent abundances of *Asplanchna*. The symbols denote the approximate morphometric relationship between *K. cochlearis* with long (LS), and short spines ﻿(﻿SS), respectively.

The monitoring data show that posterior spine length of *K*. *cochlearis* varied considerably between May and July (Fig. 2). As for the experiment, the variation in the total length of *K*. *cochlearis* shows a very similar pattern to the posterior spine length (Supporting information Fig. S2). Therefore, we here focus on the response in posterior spine length. The spine length of *K*. *cochlearis* dramatically decreased from the 8 May from 79.0 μm to 19.3 ± 7.6 μm, i.e. with 75% (Fig. 2), as newly hatched fish larvae started to feed on rotifers. Simultaneously to the reduction in spine length of *K. cochlearis*, the abundance of the predator rotifer *Asplanchna* also declined (Fig. 2), which is likely to be due to larval fish predation.

### Meta-analysis

**Figure 3:**
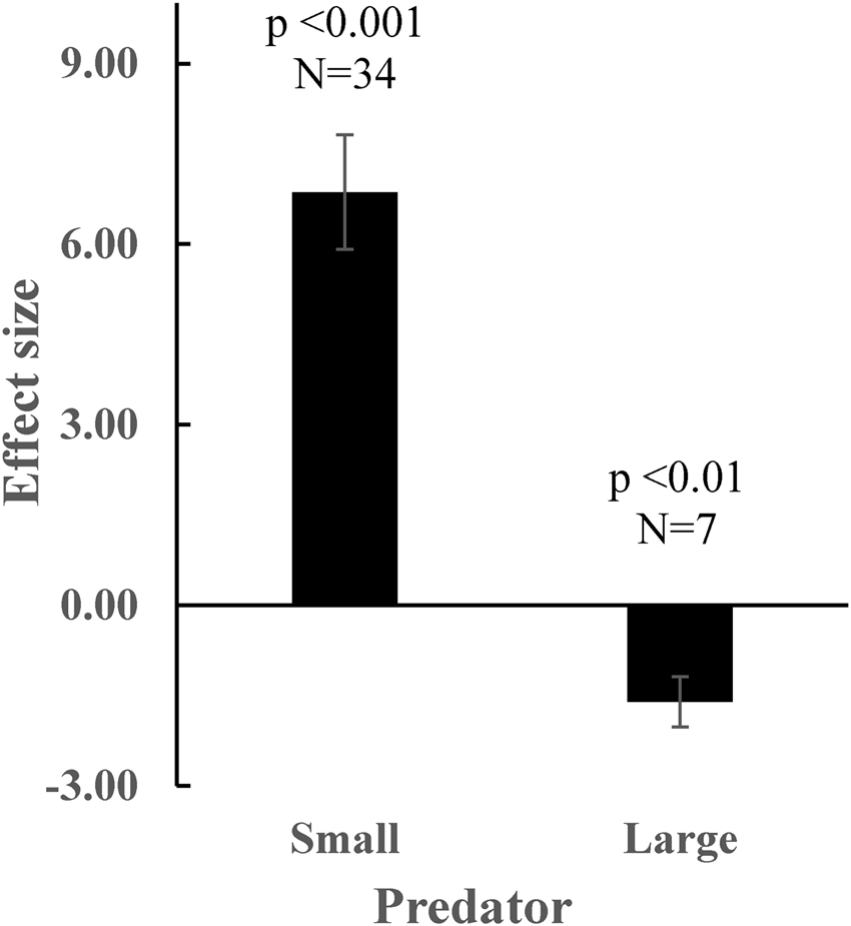
Mean effect sizes for the magnitude of induced defences for small-sized and large-sized predators. Error bars are standard errors.

Overall, the meta-analysis showed that predator kairomones induced a significant change in rotifer spine length [d = 5.39, 95% CI (3.40, 7.37), n = 41, p < 0.001)], clearly confirming that inducible plastic defensive responses to predators are present in rotifer prey. However, subgrouping analysis showed that although both small-sized and large-sized predators induced significant changes in rotifer spine length, those changes were in opposite directions. Small-sized predators significantly induced the elongation of spines in rotifer prey [d = 6.86, 95% CI (4.99, 8.73), n = 34, p < 0.001 (Fig. 3)], whereas large-sized predator induced a significant reduction [d = −1.60, 95% CI (−2.42, −0.78), n = 7, p < 0.001 (Fig. 3)]. Rosenthal’s fail-safe number for both the small-sized predator and large-sized predator groups were much larger (5465 and 86) than the critical values (180 and 45, respectively), suggesting that publication bias is unlikely to explain the observed results and therefore, the observed results are robust.

## Discussion

A major challenge for organisms in the wild is to sense and adjust to spatial and temporal changes in the threat landscape of their daily life. Predation is here a major threat since failure to adjust to changes in a predator regime will lead to a complete and immediate loss of fitness. Therefore, many prey species have evolved abilities to form predator-specific defences in order to improve their chances of survival under a predator attack^24, 25^ and such risk assessments have likely been important in the evolution of inducible defences^14^. Here, we address the morphological responses of a rotifer prey taxon to a shift in predator dominance from a small invertebrate (*Asplanchna* sp.) to a large vertebrate predator (larval fish), which occur in most aquatic ecosystems as fish larvae hatch and initiate feeding on small rotifer prey^15^. We show, experimentally, that large-sized predators induce a reduction in rotifer spine length. In a monitoring study in the field we also show that the spine length of the prey rotifer *K. cochlearis* changed, in opposite directions, in response to the shift in dominance between small-sized and large-sized predators. Hence, the monitoring data from a natural system corroborate our hypothesis that rotifer prey (*K. cochlearis*) are able to adapt their spine length, with opposing adjustment, depending on the size-specificity of the different predators they are exposed to. Furthermore, in accordance with the results from our experiment, results from a field monitoring study and a meta-analysis, covering a wide array of rotifer prey taxa, show that rotifers are able to not only sense the presence of a predator but also respond adequately to the identity of it with a bi-directional change in spine length. Hence, exposure to small-sized predators elongated rotifer spine length, whereas large-sized predators induced a reduction in prey spine length.

Previous experimental studies on rotifer predator-prey responses have mainly focused on the predatory rotifer *Asplanchna* spp. and their prey rotifers^14, 26^. *Asplanchna* is about two times larger than most rotifer prey species, such as *Brachionus calyciflorus* (the most studied rotifer prey species on inducible defences by predators), and about four times larger than *K*. *cochlearis* and is therefore here categorised as a small-sized predator. Increased spine or body size in response to predators is well documented in many prey taxa^8, 10, 12, 14^, although responses to multiple predators are typically biased towards one of the predators, i.e. prey facing combined predators do not form simple intermediate defences^6^. However, we show here that rotifers are plastic in their predator response and rapidly adjust their protective spine morphology in accordance to a wide range in predator size, taxa and feeding modes, and actually show a gradual decrease in spine length along a gradient of increasing size of predators (Fig. 1). Hence, elongated spines may make it more difficult for small predators to capture and handle the prey^27, 28^, whereas rotifers with reduced spine length (body size) may experience less predation threat from large predators, as feeding efficiency is higher on larger prey^29^.

Prey rotifers coping with small predators benefit in at least two ways from increased spine length. Firstly, increased spine length in combination with other morphological traits (e.g. stiff lorica of *Keratella*) effectively increases prey handing time^30^, thereby reducing predation rate^31^. Furthermore, the dominant rotifer predator, *Asplanchna*, prefers smaller prey which can be trapped in its pharynx, allowing rejection of prey exceeding a certain size or having spines that are too long to be swallowed^12, 32^. Thus, larger, and more spinous morphs usually are much less likely to be captured and ingested by those small predators. Hence, a small predator may selectively predate on small and short-spined individuals^33^. The generation time of *K*. *cochlearis* is only about 2–7 days^34^, suggesting that the rapid change in spine length in the wild may either be a plastic response, within an individual’s life time, or a maternal effect similar to that which has been shown for other zooplankton taxa, such as *B*. *calyciflorus* ^35^ and *Daphnia* ^36^, or due to selective predation by predators.

Although extrapolating laboratory results to complex natural ecosystems can be very challenging, patterns from field studies are indeed consistent with conclusions from the experiment. Our monitoring data show that the longer spine length of *K*. *cochlearis* co-occur with high abundances of small-sized predators (Fig. 2), which is consistent with previous studies^37^. Moreover, both Marinone and Zagarese (1991) and Baião *et al*.^38^ showed that long spine length in *K*. *tropica* and *K*. *cochlearis* were related to high density of the small-sized copepod predator *Acanthocyclops robustus* ^38, 39^. Hence, a variety of predators and ecological conditions can affect spine development in rotifers, with some predators promoting and others inhibiting spine development. Although our knowledge on which predator chemicals are active kairomones in aquatic ecosystems is scarce^40^, our results show clear evidence that different predator taxa release different kairomones and that prey rotifers use this information to distinguish between predator taxa and feeding mode and respond accordingly by adjusting their spine length. Our monitoring data show a reduction in spine length in *K*. *cochlearis* during mid-May and June, when newly hatched fish (large predators) started to feed on rotifers. In line with this, Conde-Porcuna *et al*. (1993) showed that the proportion of un-spined *K*. *cochlearis* (small size and no posterior spine) sharply increased in May and June^41^. A non-exclusive, alternative mechanism explaining the reduced spine length of *K*. *cochlearis* in natural systems may also have been predation by fish larvae on small-sized predators (e.g. *Asplanchna*), thereby releasing the rotifer prey from predation by invertebrate predators. Moreover, spine length reduction may also have been directly affected by selective predation on larger sized individuals of *K*. *cochlearis* in the field, since long-spined individuals are generally also large bodied, which has been well documented^10–12^ and confirmed by our results (Fig. 2 and Fig. S2). However, in our experimental study, we used kairomones from large predators, i.e. no selective predation occurred, and we can therefore exclude both selective predation and indirect predation on predatory rotifers as factors causing the spine length reduction in *K*. *cochlearis* in our experiment. So, the sharp reduction in *K*. *cochlearis* spine length in the lake during mid-May and June (Fig. 2) was likely driven by several co-occurring mechanisms, including elimination of small-sized predators and selective feeding on the larger-sized individuals of *K*. *cochlearis* by fish larvae, as well as fish kairomone induction of shorter spine length in *K. cochlearis*. Hence, by combining experimental results with monitoring data from a field study and a meta-analysis, we can, for the first time, conclude that rotifer prey, such as *K. cochlearis*, are able to detect and respond to differently sized consumers by adjusting their spine length in different directions.

In summary, our analyses reveal that rotifer prey can detect and respond appropriately, in opposite directions, to different sizes and feeding modes of predators; a finding that adds to the theoretical development within both predator-prey dynamics and plasticity responses to environmental conditions. The direction of the response in rotifer spine length is thus predator size specific and prey rotifers are clearly able to distinguish between kairomones released by different taxa of predators and then respond accordingly. Hence, our study provides a robust explanation to the generally observed phenomenon that many rotifer taxa differ spatially, as well as show considerable seasonal differences in spine length.

### Data availability

The datasets generated during the current study are available from the corresponding author on reasonable request.

## Electronic supplementary material

Supplementary information
