# Supplementary material for: Bi-directional plasticity: Rotifer prey adjust spine length to different predator regimes
Source: Sci Rep. 2017 Aug 31;7:10254. doi: 10.1038/s41598-017-08772-7 (PMC5579284; doi:10.1038/s41598-017-08772-7)
Supplement: Supplementary file 1 — Supplementary information [file 41598_2017_8772_MOESM1_ESM.pdf]

Supporting information

Bi-directional plasticity: Rotifer prey adjust spine length to different predator regimes

Huan Zhang, Johan Hollander, Lars-Anders Hansson

Department of Biology, Aquatic Ecology, Lund University, Lund, Sweden

Fig. S1 Total length of *Keratella cochlearis* after 12 days of exposure to kairomones from predator-free control aquaria and different predators, including the copepod (*Cyclops* sp.), the insect larvae *Chaoborus flavicans*, and small fish (*Paracheirodon innesi*). Values are means ± 1SE.

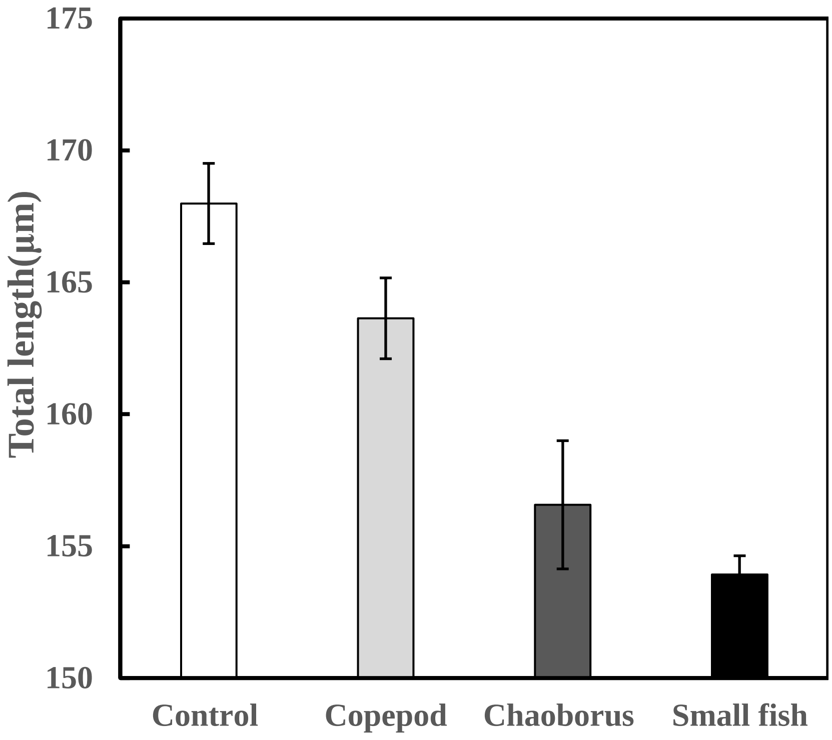

Fig. S2. Variation in total length of *Keratella cochlearis* from May to July 2013 in Lake Krankesjön. Values are means ± 1SD

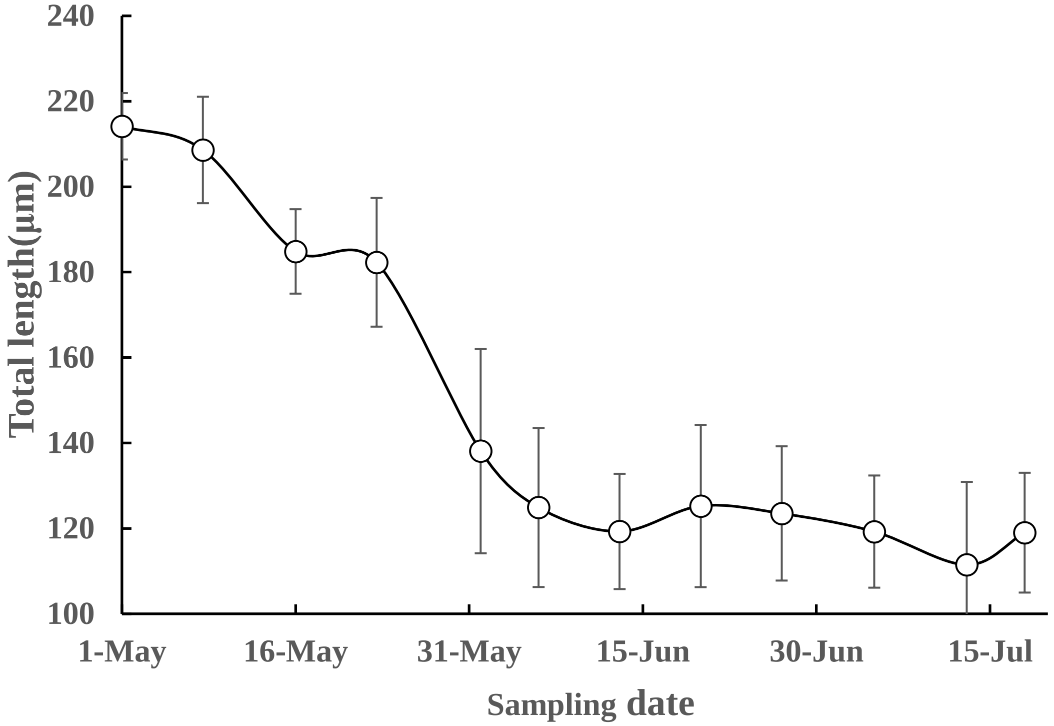

Table S1. Predator size ranges of predator groups included in the meta-analysis, and their categorisation into small and large-sized predators, respectively. The references are listed at the end of the table.

**Predator Taxa**	**Female adult size range (mm)**	**Female adult size mean (mm)**	**Predator Group**	**Reference**
**Predatory Rotifers**				
Asplanchna brightwellii	0.7 - 1.4	1.05	small-sized predator	(Wang 1961)
Asplanchna girodi	0.5 - 0.7	0.6	small-sized predator	(Wang 1961)
Asplanchnopus multiceps	0.7 - 0.88	0.79	small-sized predator	(Wang 1961)
Asplanchna priodonta	0.67 - 1.2	0.94	small-sized predator	(Wang 1961)
Asplanchna sieboldii	0.6 - 1.8	1.2	small-sized predator	(Wang 1961)
Asplanchna silvestrii	0.4 - 1.15	0.78	small-sized predator	(Koste 1978; Russell 1962)
**Copepods**				
Epischura lacustris	1.8 -2.0	1.9	small-sized predator	(Balcer et al. 1984)
Mesocyclope pehpeiensis	1.5 – 1.7	1.6	small-sized predator	(Suárez-Morales and Tqrres 2005)

Notodiaptomus incompositus	1.3	1.3	small-sized predator	(Perbiche-Neves et al. 2015)
Tropocyclops prasinus	0.5 - 0.9	0.6	small-sized predator	(Balcer et al. 1984)
Cyclops sp.	3.2-4.5	3.8	Large - sized predator	(This study)
**Others**				
Buenoa fuscipennis	4.8 - 5.4	5.1	Large - sized predator	(Heckman 2011)
Chaoborus punctipennis IV	6.5 - 8.5	7.55	Large - sized predator	(Eaton 1983)
Chaoborus flavicans	9.5-11.5	10	Large - sized predator	(This study)
Cypris pubera	2.2	2.2	Large - sized predator	(Gilbert 2012)
Paracheirodon innesi	22-24	23	Large - sized predator	(This study)
Stenostomum leucope	2.0 - 5.0	3.5	Large - sized predator	(Nandini et al. 2011)

Balcer, M. D., N. L. Korda, and S. I. Dodson. 1984. Zooplankton of the Great Lakes: a guide to the identification and ecology of the common crustacean species. Univ of Wisconsin Press, Madison, WI.

Eaton, K. A. 1983. The life history and production of *Chaoborus punctipennis* (Diptera: Chaoboridae) in Lake Norman, North Carolina, USA. Hydrobiologia **106:** 247-252.

Gilbert, J. J. 2012. Effects of an ostracod (*Cypris pubera*) on the rotifer *Keratella tropica*: predation and reduced spine development. International Review of Hydrobiology **97:** 445-453.

Heckman, C. W. 2011. Encyclopedia of South American Aquatic Insects: Hemiptera-Heteroptera: Illustrated Keys to Known Families, Genera, and Species in South America. Springer Science & Business Media. Dordrecht, Heidelberg, London, New York

Koste, W. 1978. Rotatoria: die radertiere mitteleuropas. Rotatoria: die radertiere mitteleuropas. Gebruder Borntraeger, Berlin, Stuttgart.

Nandini, S., S. Sarma, and H. J. Dumont. 2011. Predatory and toxic effects of the turbellarian (*Stenostomum cf leucops*) on the population dynamics of *Euchlanis dilatata*, *Plationus patulus* (Rotifera) and *Moina macrocopa* (Cladocera). Hydrobiologia **662:** 171-177.

Perbiche-Neves, G., G. A. Boxshall, D. Previattelli, M. G. Nogueira, and C. E. F. Da Rocha. 2015. Identification guide to some Diaptomid species (Crustacea, Copepoda, Calanoida, Diaptomidae) of "de la Plata" River Basin (South America). ZooKeys**:** 1-111.

Russell, C. 1962. Additions to the Rotatoria of New Zealand, Part IX, p. 337-341. Transactions of the Royal Society of New Zealand.

Suárez-Morales, E., and J. L. Tqrres. 2005. The Asian *Mesocyclops pehpeiensis* Hu, 1943 (Crustacea, Copepoda, Cyclopidae). Zoosystema **27**.

Wang, J. 1961. Freshwater Rotifer Fauna in China. Science Press, Beijing, China.

Table S2. List of predators, prey, individual effect sizes (*d*) and references reviewed in the meta- analysis. Predator size group includes Small-sized (SP) and Large sized (LP) predators. The ID number refers to references given at the end of the table.

Predator	Prey	Predator size group	Predator taxa	Variable	*d*	ID
Asplanchna brightwellii	*Brachionus calyciflorus*	*SP*	*Rotifera*	spine length	8.81	1
Asplanchna brightwellii	*Brachionus calyciflorus(LY)*	*SP*	*Rotifera*	spine length	24.79	2
Asplanchna brightwellii	*Brachionus calyciflorus(BJ)*	*SP*	*Rotifera*	spine length	31.51	2
Stenostomum leucope	*Brachionus havanaensis*	*LP*	*flatworm*	spine length	-1.62	3
Asplanchna girodi	*Brachionus havanaensis*	*SP*	*Rotifera*	spine length	1.02	3
Mesocyclops pehpeiensis	*Brachionus havanaensis*	*SP*	*Copepoda*	spine length	-1.36	3
Asplanchna girodi	*Brachionus variabilis*	*SP*	*Rotifera*	spine length	11.01	4
Asplanchna brightwellii	*Brachionus calyciflorus*	*SP*	*Rotifera*	spine length	21.34	5
Asplanchna brightwellii	*Keratella tropica*	*SP*	*Rotifera*	spine length	10.42	5
Asplanchna brightwelli	*Keratella tropica*	*SP*	*Rotifera*	spine length	151.64	6
Asplanchna brightwelli	*Brachionus calyciflorus*	*SP*	*Rotifera*	spine length	9.46	6
Asplanchna brightwelli	*Brachionus calyciflorus Pallas*	*SP*	*Rotifera*	spine length	17.68	7

Asplanchna brightwelli	*Plationus macracanthus*	*SP*	*Rotifera*	spine length	4.26	7
Asplanchna brightwelli	*Keratella tropica*	*SP*	*Rotifera*	spine length	9.79	8
Asplanchna sieboldii	*Brachionus calyciflorus*	*SP*	*Rotifera*	spine length	2.29	9
Asplanchna brightwelli	*Brachionus calyciflorus*	*SP*	*Rotifera*	spine length	2.77	9
Asplanchna girodi	*Brachionus calyciflorus*	*SP*	*Rotifera*	spine length	1.12	9
Asplanchna brightwelli	*Brachionus calyciflorus*	*SP*	*Rotifera*	spine length	11.87	10
Asplanchna brightwelli	*Brachionus havanaensis*	*SP*	*Rotifera*	spine length	13.95	10
Asplanchnopus multiceps	*Lecane stokesii*	*SP*	*Rotifera*	spine length	1.30	11
Asplanchna girodi	*Brachionus havanaensis*	*SP*	*Rotifera*	spine length	6.44	12
Asplanchna girodi	*Brachionus quadridentatus*	*SP*	*Rotifera*	spine body ratio	4.49	13
Asplanchna brightwelli	*Brachionus calyciflorus pala*	*SP*	*Rotifera*	spine length	4.86	14
Asplanchna brightwelli	*Brachionus calyciflorus dorcas*	*SP*	*Rotifera*	spine length	6.41	14
Asplanchna sieboldii	*Brachionus calyciflorus Pallas*	*SP*	*Rotifera*	spine length	7.06	15
Asplanchna girodi	*Keratella slacki*	*SP*	*Rotifera*	spine length	2.65	16
Tropocyclops prasinus	*Keratella slacki*	*SP*	*Copepoda*	spine body ratio	-0.15	16
Tropocyclops prasinus	*Keratella cochleaari*	*SP*	*Copepoda*	spine length	3.92	17

Asplanchna girodi	*Keratella testudo*	*SP*	*Rotifera*	spine length	8.86	18
Asplanchna brightwelli	*Keratella testudo*	*SP*	*Rotifera*	spine length	7.75	18
Asplanchna silvestrii	*Keratella testudo*	*SP*	*Rotifera*	spine length	4.53	18
Asplanchna priodonta	*Keratella testudo*	*SP*	*Rotifera*	spine length	12.13	18
Epischura lacustris	*Keratella testudo*	*SP*	*Copepoda*	spine length	4.28	18
Chaoborus punctipennis	*Keratella testudo*	*LP*	*Insecta*	spine length	0	18
Cypris pubera	*keratella tropica*	*LP*	*Ostracoda*	spine length	-1.68	19
Asplanchna brightwelli	*Keratella tropica*	*SP*	*Rotifera*	spine length	31.95	20
Notodiaptomus incompositus	*Keratella tropica*	*SP*	*Copepoda*	spine length	4.44	21
Buenoa fuscipennis	*Keratella tropica*	*LP*	*Insecta*	spine length	-18.20	21
Paracheirodon innesi	*Keratella cochlearis*	*LP*	*Fish*	spine length	-3.58	This study
Chaoborus flavicans	*Keratella cochleaari*	*LP*	*Insecta*	spine length	-1.77	This study
Cyclops sp.	*Keratella cochleaari*	*LP*	*Copepoda*	spine length	-0.81	This study

List of references include in the meta-analysis.

1. Yin, X. W., Y. C. Zhou, X. C. Li, and W. X. Li. 2015. Reduced investment in sex as a cost of inducible defence in *Brachionus calyciflorus* (Rotifera). Freshwater Biology **60**:89–100

2. Yin, X. W., N. X. Zhao, B. H. Wang, W. J. Li, and Z. N. Zhang. 2014. Transgenerational and within-generational induction of defensive morphology in *Brachionus calyciflorus* (Rotifera): importance of maternal effect. Hydrobiologia **742**:313-325.

3. Nandini, S., F. S. Zuniga-Juarez, and S. S. S. Sarma. 2014. Direct and indirect effects of invertebrate predators on population level responses of the rotifer *Brachionus havanaensis* (Rotifera). International Review of Hydrobiology **99**:107-116.

4. Gilbert, J. J. 2014. Morphological and behavioral responses of a rotifer to the predator *Asplanchna*. Journal of Plankton Research **36**:1576-1584.

5. Gilbert, J. J. 2012b. Predator-induced defense in rotifers: developmental lags for morph transformations, and effect on population growth. Aquatic Ecology **46**:475-486.

6. Gilbert, J. J. 2011a. Induction of different defences by two enemies in the rotifer *Keratella tropica*: response priority and sensitivity to enemy density. Freshwater Biology **56**:926-938.

7. Sarma, S., R. A. L. Resendiz, and S. Nandini. 2011. Morphometric and demographic responses of brachionid prey (*Brachionus calyciflorus* Pallas and *Plationus macracanthus* (Daday)) in the presence of different densities of the predator *Asplanchna brightwellii* (Rotifera: Asplanchnidae). Hydrobiologia **662**:179-187.

8. Gilbert, J. J. 2011b. Temperature, kairomones, and phenotypic plasticity in the rotifer *Keratella tropica* (Apstein, 1907). Hydrobiologia **678**:179-190.

9. Gama-Flores, J. L., M. E. Huidobro-Salas, S. Sarma, and S. Nandini. 2011. Effects of predator (*Asplanchna*) type and density on morphometric responses of *Brachionus calyciflorus* (Rotifera). Allelopathy Journal **27**:289-300.

10. Aránguiz-Acuña, A., R. Ramos-Jiliberto, and R. O. Bustamante. 2011. Experimental assessment of interaction costs of inducible defenses in plankton. Journal of Plankton Research **33**:1445-1454.

11. Soto, C. S., and S. S. S. Sarma. 2009. Morphometric changes in *Lecane stokesii* (Pell, 1890) (Rotifera: Lecanidae) induced by allelochemicals from the predator *Asplanchnopus multiceps* (Schrank, 1793). Allelopathy Journal **24**:215-221.

12. Pavón-Meza, E.L., S. Sarma, and S. Nandini, Combined effects of temperature, food (*Chlorella vulgaris*) concentration and predation(*Asplanchna girodi*) on the morphology of *Brachionus havanaensis* (Rotifera). Hydrobiologia, 2007. 593(1): p. 95-101.

13. Gilbert, J. J. 2001. Spine development in *Brachionus quadridentatus* from an Australian billabong: genetic variation and induction by *Asplanchna*. Hydrobiologia **446/447**:19-28.

14. Gilbert, J. J., and J. K. Waage. 1967. *Asplanchna*, *Asplanchna*-substance, and posterolateral spine length variation of the rotifer *Brachionus calyciflorus* in a natural environment. Ecology **48**:1027-1031.

15. Gilbert, J. J. 1967. Asplanchna and postero-lateral spine production in *Brachionus calyciflorus*. Archiv Fur Hydrobiologie **64**:1-62.

16. Gilbert, J. J., and R. S. Stemberger. 1984. *Asplanchna*-induced polymorphism in the rotifer *Keratella slacki*. Limnology and Oceanography **29**:1309-1316.

17. Stemberger, R., and J. Gilbert. 1984. Spine development in the rotifer *Keratella cochlearis*: induction by cyclopoid copepods and *Asplanchna*. Freshwater Biology **14**:639-647.

18. Stemberger, R. S., and J. J. Gilbert. 1987. Multiple-species induction of morphological defenses in the rotifer *Keratella testudo*. Ecology **68**:370-378.

19. Gilbert, J. J. 2012a. Effects of an ostracod (*Cypris pubera*) on the rotifer *Keratella tropica*: predation and reduced spine development. International Review of Hydrobiology **97**:445-453.

20. Gilbert, J. J. 2009. Predator-specific inducible defenses in the rotifer *Keratella tropica*. Freshwater Biology **54**:1933-1946.

21. Zagarese, H. E., and M. C. Marinone. 1992. Induction and inhibition of spine development in the rotifer *Keratella tropica*. Freshwater Biology **28**:289-300.
